# Inhibition of TRADD ameliorates chondrocyte necroptosis and osteoarthritis by blocking RIPK1-TAK1 pathway and restoring autophagy

**DOI:** 10.1038/s41420-023-01406-0

**Published:** 2023-03-31

**Authors:** Kai Sun, Zhou Guo, Jinming Zhang, Liangcai Hou, Shuang Liang, Fan Lu, Genchun Wang, Jingting Xu, Xiong Zhang, Fengjing Guo, Wentao Zhu

**Affiliations:** Department of Orthopedics, Tongji Hospital, Tongji Medical College, Huazhong University of Science and Technology, Wuhan, Hubei 430030 China

**Keywords:** Osteoarthritis, Chronic inflammation

## Abstract

Osteoarthritis (OA) is an age-related disease characterized by cartilage degeneration. TNFR1-associated death domain protein (TRADD) is a key upstream molecule of TNF-α signals but its role in OA pathogenesis is unknown. This study aimed to verify that whether inhibition of TRADD could protect against chondrocyte necroptosis and OA, and further elucidate the underlying mechanism. We demonstrated that TNF-α-related OA-like phenotypes including inflammation response, extracellular matrix degradation, apoptosis, and necroptosis in chondrocytes were inhibited by TRADD deficiency. Furthermore, TRADD interacted with TRAF2 and knockdown of TRADD suppressed the activation of RIPK1-TAK1-NF-κB signals and restored impaired autophagy. ICCB-19, the selective inhibitor of TRADD, also attenuated necroptosis in chondrocytes. Mechanismly, ICCB-19 blocked the phosphorylation of TAK1-NF-κB signals and restored impaired autophagy, whereas inhibiting autophagic process with 3-Methyladenine compromised these effects of ICCB-19. The in vivo study showed that the intra-articular injection of ICCB-19 rescued the expression of collagen alpha-1(II) chain and LC3, and mitigated the cartilage degeneration of OA mice. This study demonstrates that TRADD mediates TNF-α-induced necroptosis and OA-like phenotypes of chondrocytes and suggests that ICCB-19 suppresses chondrocyte damage and cartilage degeneration by inhibiting TNF-α-TRADD-mediated signals and dysregulation of autophagy in chondrocytes. ICCB-19 may serve as an important option for OA therapy.

## Introduction

Osteoarthritis (OA) is a common disease that causes physical pain and even disability in patients. This disease affects the majority of older people in the world and brings huge economic burden to society [1, 2]. A variety of risk factors have been identified for OA [3], but the clear pathogenesis and effective treatment remain to be further studied. The pathological changes of OA are characterized by chondrocyte death and cartilage destruction, synovitis, and osteophyte formation [4]. Chondrocytes and their secreted extracellular matrix (ECM) are main components of articular cartilage [5]. Chondrocytes maintain physiological structure of articular cartilage and improve joint function by well-regulating the balance of anabolism and catabolism of ECM [4]. Tumor necrosis factor-α (TNF-α) is a key inflammatory mediator in OA progression and one of the leading cytokines giving rise to the imbalance of matrix synthesis and decomposition [6, 7]. Therefore, it is important to maintain homeostasis of chondrocytes by protecting chondrocytes against death and degradation of ECM under the pathological conditions.

TNFR1-associated death domain protein (TRADD), is an adaptor molecule for the signaling downstream of tumor necrosis factor receptor 1 (TNFR1) [8, 9]. When TNFR1 is bound to TNF-α, TRADD can be recruited to TNFR1, and undergoes a conformational change, enable it to recruit two other intracellular adaptor proteins receptor-interacting protein kinase 1 (RIPK1) and tumor necrosis factor receptor-associated factor 2 (TRAF2), which form a complex (complex I) near the cytomembrane [10]. Then complex I can activate the nuclear factor kappa-B (NF-κB) pathway [11]. The TRADD containing signalosome can be modified by recruitment of another protein called Fas-associated death domain (FADD). FADD converts the signalosome to a death-inducing signaling complex, complex 2, that can activate pro-caspase 8, which in turn activates caspase 3 to initiate apoptosis [12]. However, when caspase-8 or FADD is deficient, cells are unable to undergo apoptosis but rather are susceptible to another form of TNF-induced cell death called necroptosis [13, 14]. Although the signaling role of TRADD has been well elucidated, its role in OA progression remains unclear.

ICCB-19 is a novel compound that can specifically bind to the N-terminal of TRADD, thereby inhibiting the function of TRADD and formation of complex I [9]. Therefore, it is speculated in this study that ICCB-19 may also play a role in chondrocyte damage and cartilage degeneration via inhibiting TRADD. In present study, we examined the expression of TRADD in chondrocytes and OA cartilage. We sought to verify the effect of TRADD and ICCB-19 in TNF-α-induced OA-like phenotypes and surgery-induced cartilage degeneration and further elucidate the underlying mechanism.

## Results

### TNF-α could induce OA-like phenotypes in chondrocytes

**Fig. 1:**
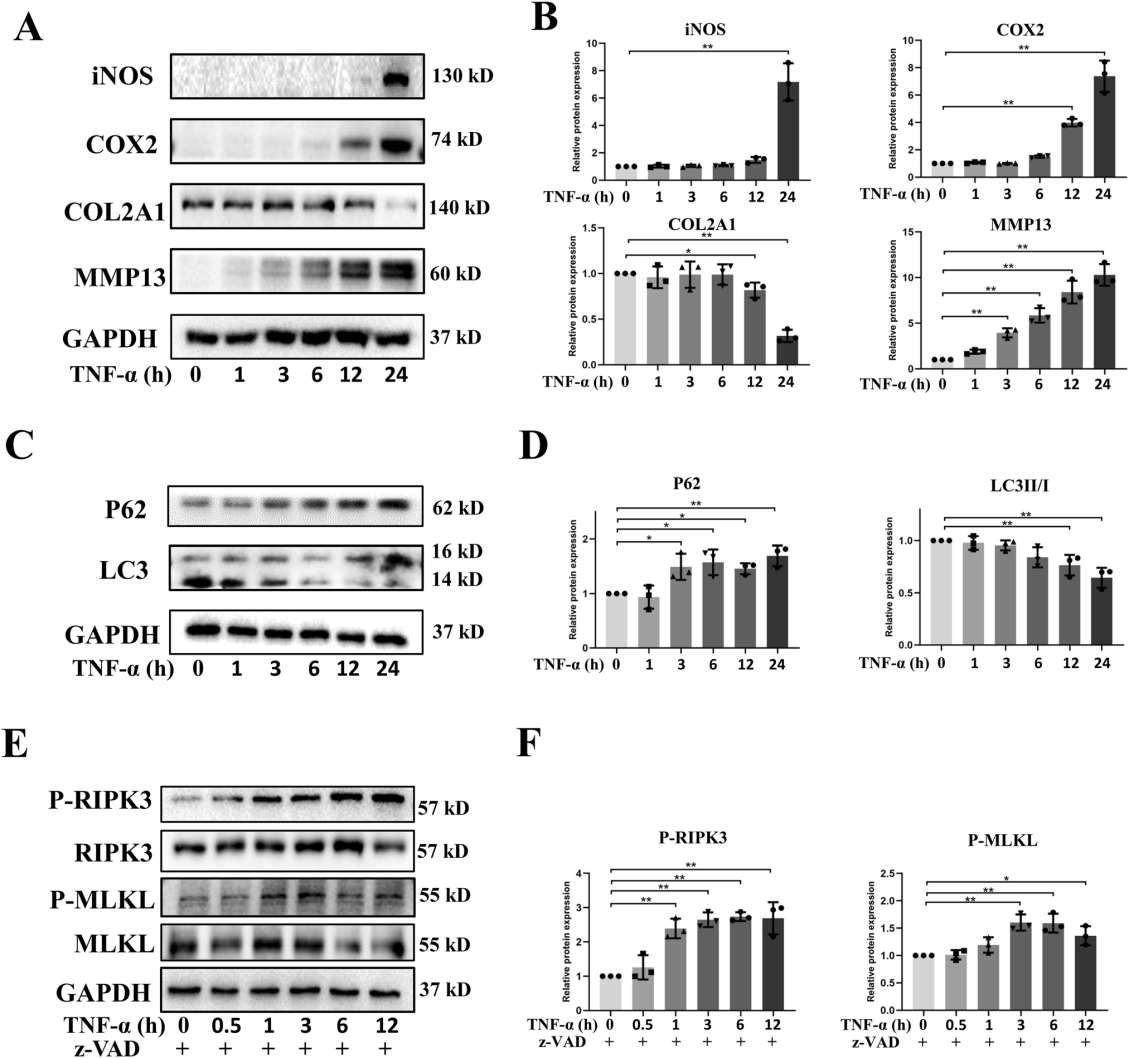
TNF-α-mediated cellular events in chondrocytes. Chondrocytes were treated with TNF-α (5 ng/ml) for 0, 1, 3, 6, 12, and 24 h, the protein expression of iNOS, COX2, COL2A1, MMP13, P62, and LC3 was examined (**A**, **C**). Chondrocytes were pre-treated with z-VAD for 2 h and then exposed to TNF-α for 0, 0.5, 1, 3, 6, and 12 h respectively, the phosphorylation of RIPK3 and MLKL was detected using western blot (**E**). (**B**, **D**, **F**) Quantitative analysis of protein bands by image J and data in figures are expressed as means ± SD. *p < 0.05, **p < 0.01. C-caspase-3, cleaved-caspase-3. z-VAD, Z-VAD-FMK.

TNF-α is a key inflammatory mediator implicated in cartilage degeneration [6]. We investigated the detrimental effects of TNF-α on primary chondrocytes. The results showed that TNF-α (5 ng/ml) could induce the increased expression of inflammatory markers, inducible nitric oxide synthase (iNOS) and cyclooxygenase-2 (COX2), and promoted the expression of matrix metallopeptidase 13 (MMP13), a catabolic marker of ECM (Fig. 1A, B). Whereas TNF-α inhibited the expression of Collagen alpha-1(II) chain (COL2A1) (Fig. 1A, B). Microtubule-associated protein 1 light chain 3 (LC3) and P62 were important for autophagy process [15]. We found that TNF-α decreased the ratio of LC3II/I while promoted the expression of P62, indicating reduction of autophagy (Fig. 1C, D). Additionally, when combined with z-VAD-FMK, a caspase inhibitor, TNF-α also induced the increased phosphorylation of receptor interacting serine/threonine kinase 3 (RIPK3) and mixed lineage kinase domain like pseudokinase (MLKL) (Fig. 1E, F), suggesting that TNF-α and z-VAD-FMK could also cause necroptosis of chondrocytes. These results demonstrated that TNF-α was involved in cartilage degeneration in terms of inflammatory responses, imbalanced metabolism of ECM, impaired autophagy level, and necroptosis in chondrocytes.

### TRADD is highly expressed in OA cartilage

**Fig. 2:**
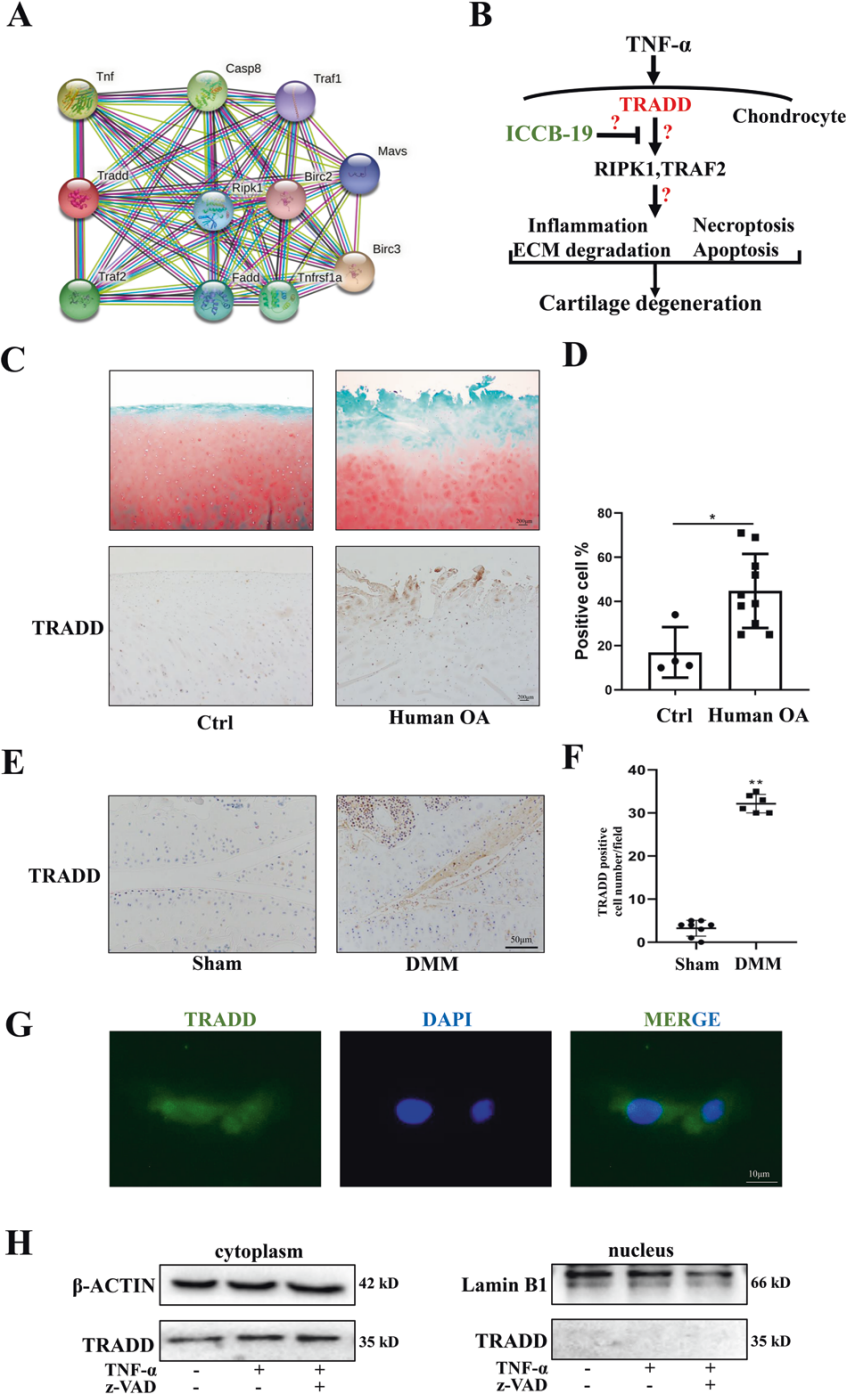
TRADD is highly expressed in OA cartilage. **A** The relationship and interaction between TRADD and other molecules shown in STRING database. **B** The hypothesis diagram of this study. **C**, **D** Representative images of Safranin O/Fast green staining and immunohistochemistry staining of TRADD-positive chondrocytes in human knee cartilage (scale bar: 200 μm) and statistical analysis of the ratio of TRADD-positive cells in control group (*n* = 4) and OA group (*n* = 10). **E**, **F** Representative images of immunohistochemistry staining of TRADD-positive chondrocytes (scale bar: 50 μm) and statistical analysis of the ratio of TRADD-positive cells in Sham group (*n* = 8) and DMM group (*n* = 6). **G** Representative images of immunofluorescence staining of TRADD-positive chondrocytes, scale bar: 10 μm. **H** Chondrocytes were stimulated by TNF-α for 24 h with or without z-VAD, TRADD in cytoplasm and nucleus were detected by western blot respectively. The data represents the means ± S.D. Significant difference between the DMM and sham groups are indicated as *P < 0.05, **P < 0.01.

To find out key fortress of TNF-α signaling, we searched STRING database and TRADD is labeled as a core upstream molecule in TNF-α-related pathway (Fig. 2A). However, the role of TNF-α-TRADD in chondrocytes and cartilage degeneration and underlying mechanism remain unclear (Fig. 2B). Therefore, we firstly investigated its expression in cartilage and chondrocytes using immunohistochemistry and immunofluorescence. TRADD expression was upregulated in cartilage of patients with OA compared to the control group (Fig. 2C, D). In addition, a significant higher rate of TRADD-positive cell was found in cartilage of DMM mice compared to the sham group (Fig. 2E, F). At cellular level, the immunofluorescence results showed that TRADD was expressed at a low level in untreated chondrocytes and mainly distributed in cytoplasm of chondrocyte (Fig. 2G). Moreover, the detection of nucleoprotein and cytoplasmic protein also confirmed that TRADD was mainly distributed in the cytoplasm, and TNF-α or TNF-α + z-VAD treatment did not change the distribution of TRADD (Fig. 2H). These evidence indicates that TRADD is highly expressed in OA cartilage and may play a role in chondrocyte and cartilage degeneration.

### TRADD mediates TNF-α-related damage in chondrocytes

**Fig. 3:**
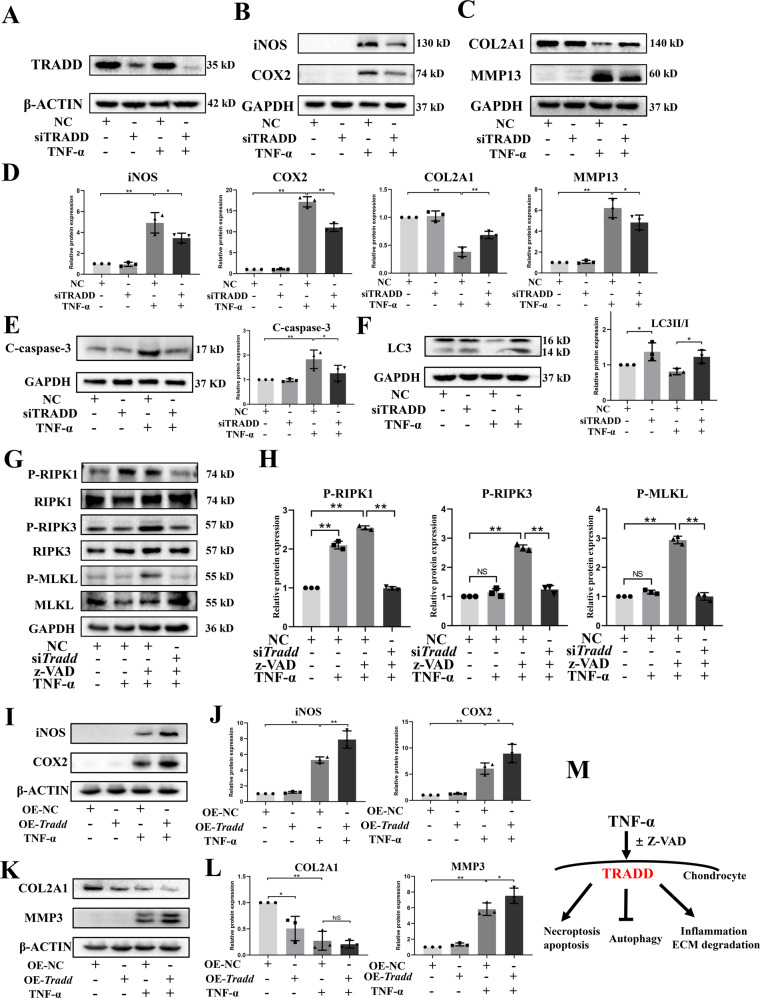
The role of TRADD in TNF-α-mediated cellular events. The cells were transfected with siRNA and lipofectamine 3000 for 48 h. After 48 h transfection, the medium was replaced by refresh medium and chondrocytes were treated with TNF-α (5 ng/ml) for 24 h, the protein expression of TRADD, iNOS, COX2, COL2A1, MMP13, cleaved-caspase-3, and LC3 was examined (**A**–**C**, **E**, **F**). After 48 h transfection with siRNA and lipofectamine 3000, the medium was replaced by refresh medium and chondrocytes were pre-treated with z-VAD for 2 h and then exposed to TNF-α for 24 h, the phosphorylation of RIPK1, RIPK3, and MLKL as well as protein expression of these three markers were detected using western blot (**G**). **I**, **K** The protein expression of iNOS, COX2, COL2A1, and MMP3 was examined in chondrocytes overexpressing TRADD after TNF-α intervention. **D**, **H**, **J**, **L** Semi-quantitative analysis of protein bands by image J and data in figures were expressed as means ± SD. *p < 0.05, **p < 0.01. NS not significant,. NC negative control. z-VAD, Z-VAD-FMK. C-caspase-3, cleaved-caspase-3. **M** The graphical scheme summarized the findings of the role of TRADD in TNF-α-mediated chondrocytes events.

Furthermore, the effect of TRADD in chondrocytes were investigated. The expression of TRADD was successfully downregulated by small interfering RNA (Fig. 3A), and downregulating TRADD in TNF-α stimulated chondrocyte significantly reduced the expression of iNOS, COX2, MMP13, whereas enhanced the expression of COL2A1 compared to TNF-α-treated group (Fig. 3B–D). Moreover, Knockdown of TRADD repressed the protein level of TNF-α-induced cleaved-caspase-3, indicating the reduced apoptosis of chondrocytes (Fig. 3E). Interestingly, TNF-α downregulated the ratio of LC3II/I, which were also reversed by the knockdown of TRADD (Fig. 3F). Furthermore, chondrocyte necroptosis was induced by combined treatment of TNF-α and z-VAD-FMK and the effect of TRADD in chondrocyte necroptosis was investigated. The data revealed that deficiency of TRADD also significantly inhibited activation of RIPK1, RIPK3, and MLKL, evident by reduced phosphorylation of these proteins (Fig. 3G, H). Contrarily, overexpression of TRADD further promoted the expression of iNOS, COX2, and MMP3 in TNF-α-treated chondrocytes and inhibited the protein level of COL2A1 (Fig. 3I–L). These data indicated that TRADD is involved in TNF-α-related damage of chondrocytes and downregulating TRADD can protect chondrocytes against TNF-α-mediated inflammation response, ECM degradation, impaired autophagy, and necroptosis (Fig. 3M).

### TRADD mediates TNF-α-TRAF2-TAK1-NF-κB pathway in chondrocytes

**Fig. 4:**
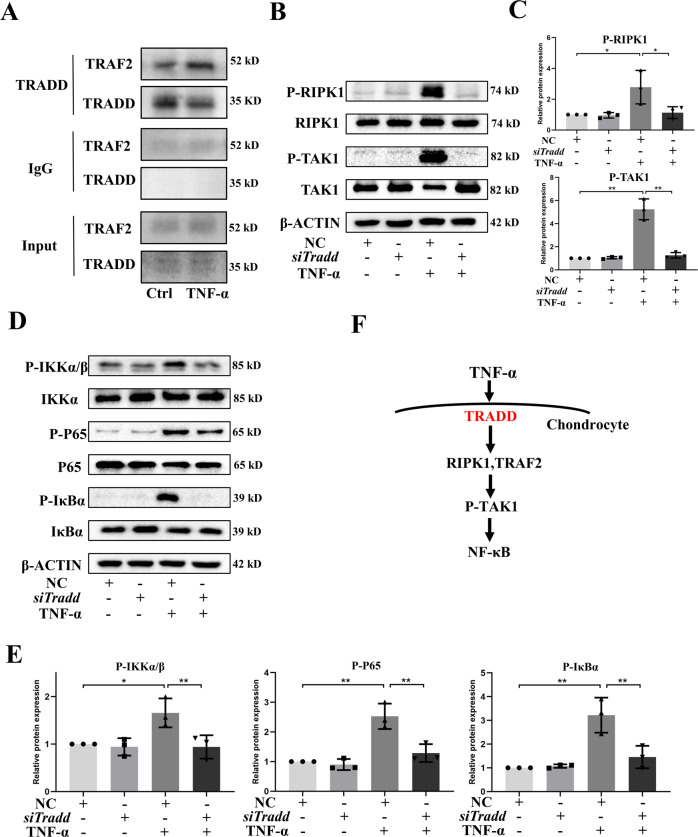
TRADD mediates TNF-α-TRAF2-TAK1-NF-κB pathway in chondrocytes. Chondrocytes were treated with TNF-α (5 ng/ml) for 15 min. **A** The binding between TRADD and TRAF2 was identified by Co-IP experiment, the group added with IgG was defined as negative control. **B**, **D** After the transfection of siRNA and TNF-α treatment, the phosphorylation of RIPK1, TAK1, IKKα/β, IκBα, and P65 were detected by western blot. **C**, **E** Semi-quantitative analysis of protein bands by image J and data in figures were expressed as means ± SD. *p < 0.05, **p < 0.01. NS not significant. NC negative control. **F** The schematic diagram of TRADD-mediated pathway in chondrocytes.

Our previous study indicated that RIPK1-TRAF2-NF-κB axis in chondrocyte was involved in cartilage degeneration [16] and it was reported that TRADD could interact with TRAF2 and RIPK1 [17]. Therefore, we further investigated whether this axis mediates the effects of TRADD in chondrocytes. The Co-IP result revealed that TRADD could bind with TRAF2 in chondrocytes, indicating that TRAF2 may be involved in the function of TRADD (Fig. 4A). We further knocked-down the TRADD expression and found that the activation of RIPK1 induced by TNF-α was attenuated (Fig. 4B, C). Furthermore, the canonical downstream effector of TNF-α signal, transforming growth factor beta-activated kinase 1(TAK1)-NF-κB were also examined and, interestingly, the phosphorylation of TAK1, IκB kinaseα/β (IKKα/β), inhibitor of NF-κB α (IκBα), and P65 provoked by TNF-α were inhibited by knockdown of TRADD (Fig. 4D, E). These data suggested that TRADD mediates TNF-α-TRAF2-TAK1-NF-κB pathway in chondrocytes (Fig. 4F).

### ICCB-19 is a key inhibitor of TRADD pathway in chondrocytes

**Fig. 5:**
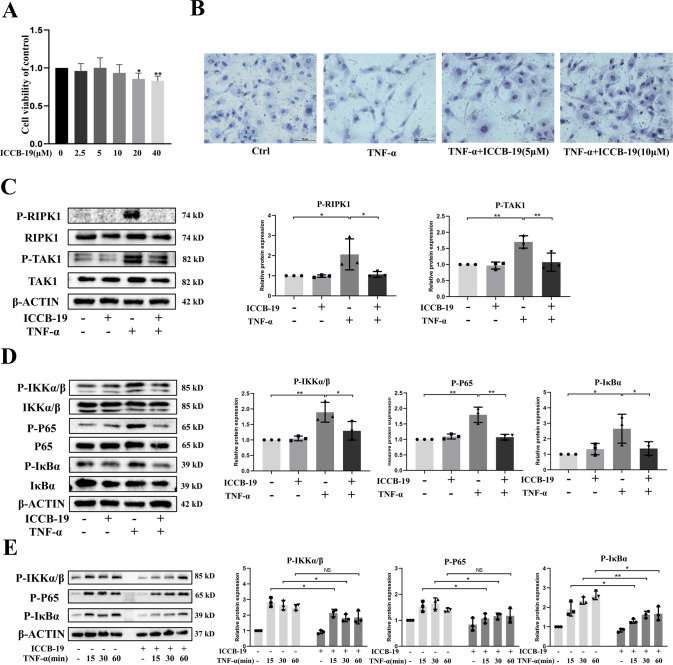
The inhibitory effect of ICCB-19 for TRADD-mediated signals. **A** Cell viability of chondrocytes exposed to ICCB-19 for 24 h was determined by CCK-8 assay. **B** The morphology of chondrocytes was revealed by toluidine blue staining, scale bar:100 μm. **C**, **D** Chondrocytes were pre-treated ICCB-19 for 2 h and then exposed to TNF-α for 15 min, the phosphorylation of RIPK1, TAK1, IKKα/β, IκBα, and P65 were detected by western blot. **E** After chondrocytes were treated by TNF-α with or without ICCB-19 at time points of 15, 30, and 60 min, the changes of phosphorylation of IKKα/β, IκBα, and P65 were detected by western blot. The semi-quantitative analysis of protein bands was executed by image J and data in figures were expressed as means ± SD. *p < 0.05, **p < 0.01. NS not significant.

Considering the vital effects of TRADD in chondrocytes, the therapeutic intervention for targeting TRADD may protect against cartilage degeneration. Of note, ICCB-19 has been reported to specifically bind to TRADD and then modulate the downstream pathway [9]. More importantly, ICCB-19 could be easily dissolved in water. Therefore, we emphatically investigated the effects of ICCB-19 on chondrocytes and cartilage degeneration. Firstly, the cellular toxicity of ICCB-19 was examined and the data showed that a low dose of ICCB-19 (2.5, 5, and 10 μM) has no effect on cell viability (Fig. 5A). In addition, the treatment of ICCB-19 (concentrations of 5 and 10 μM) partially rescued the altered morphology of chondrocytes caused by TNF-α, as revealed by toluidine blue staining (Fig. 5B). Compared with the control group, the chondrocytes treated with ICCB-19 (5 and 10 μM) showed little loss of coloration and their morphology changed from cobble-like to narrow or polygonal. To confirm whether ICCB-19 can be used as a specific inhibitor of TRADD-mediated signals, the RIPK1-TAK1-NF-κB pathway that could be modulated by TRADD was firstly investigated. The results showed that ICCB-19 (10 μM) treatment effectively repressed TNF-α-provoked phosphorylation of RIPK1, TAK1, IKKα/β, IκBα, and P65 (Fig. 5C, D), suggesting that ICCB-19 could effectively inhibit the function of TRADD, while did not affect the expression of TRADD (the data was not shown). Furthermore, the time course examination of NF-κB (time points of 15, 30, and 60 min) confirmed inhibitory effect of ICCB-19 on NF-κB signal (Fig. 5E). Collectively, these data demonstrated that ICCB-19 could inhibit the RIPK1-TAK1-NF-κB signal, indicating modulatory role of ICCB-19 in TRADD pathway.

### ICCB-19 protect chondrocytes against TNF-α-induced detrimental events

**Fig. 6:**
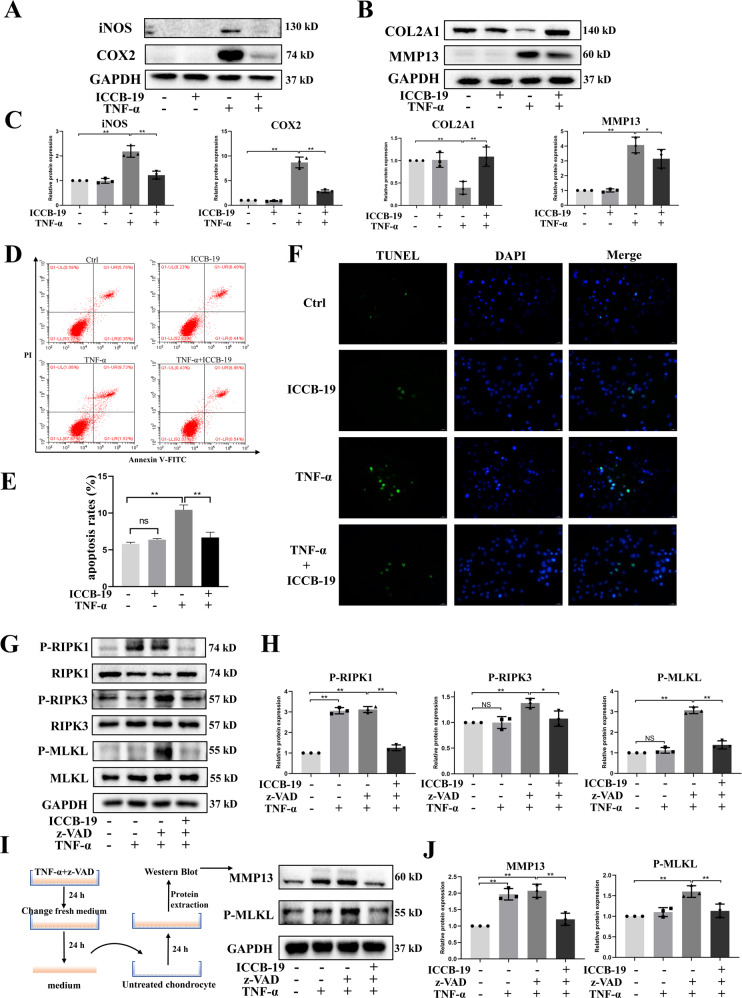
The protective effects of ICCB-19 on chondrocytes. After a pre-treatment of ICCB-19 for 2 h, chondrocytes were stimulated by TNF-α for 24 h. **A**, **B** the protein expression of iNOS, COX2, COL2A1, and MMP13 were examined. **C** The semi-quantitative analysis of protein expression. **D** The apoptotic chondrocytes were stained by Annexin V-FITC /PI and analyzed by flow cytometry. The average apoptotic rates were shown in (**E**). **F** The apoptotic chondrocytes were labeled by TUNEL staining, scale bar: 50 μm. **G** After the indicated treatment, the phosphorylation of RIPK1, RIPK3, and MLKL as well as protein expression of these three markers were detected by western blot. **H** The semi-quantitative analysis of protein expression. **I** After the induction of necroptosis, the supernatant containing TNF-α and z-VAD was replaced by refresh culture medium. 24 h later, the culture medium was transferred to untreated primary chondrocytes and culture for 24 h. The protein expression of MMP13 and phosphorylation of MLKL were examined. **J** The semi-quantitative analysis of protein expression. Data are expressed as means ± SD. *p < 0.05, **p < 0.01. NS not significant. z-VAD, Z-VAD-FMK. P-, Phosphorylated-.

To comprehensively evaluate the protective effect of ICCB-19 in chondrocytes, we investigate the effect of ICCB-19 on multiple cellular events in aspect of inflammatory response, ECM catabolism, apoptosis, and necroptosis. Our results showed that ICCB-19 (10 μM) treatment significantly reversed the upregulated expression of iNOS, COX2, and MMP13, and meanwhile restored decreased expression of COL2A1 (Fig. 6A–C). Besides, TNF-α-induced apoptosis of chondrocytes was notably attenuated by ICCB-19 treatment, as shown by Annexin V-FITC /PI staining and TUNEL staining (Fig. 6D–F). Next, the necroptosis was induced by combined treatment of TNF-α and z-VAD, and the anti-necroptotic effect of ICCB-19 was investigated. The data revealed that combined treatment of TNF-α and z-VAD enhanced the phosphorylation of RIPK1, RIPK3, and MLKL, whereas these effects were significantly inhibited by ICCB-19 treatment (Fig. 6G, H). Necroptotic cell is characterized by the disruption of the plasma membrane and release of cell content [18]. Therefore, we conducted an indirect co-culture experiment to verify the effect of necroptosis and ICCB-19. After the induction of necroptosis, the supernatant containing TNF-α and z-VAD was replaced by refresh culture medium. 24 h later, the culture medium was transferred to untreated primary chondrocytes. The results showed the culture medium also induced upregulation of MMP13 expression and the activation of MLKL, further confirming the occurrence of necroptosis in chondrocytes. Interestingly, ICCB-19 still reversed these effects (Fig. 6I, J). The above evidence demonstrated that ICCB-19 could protect chondrocytes against TNF-α-induced detrimental events including inflammatory response, ECM degradation, apoptosis, and necroptosis.

### Autophagy mediates protective role of ICCB-19 in TNF-α-induced detrimental events

**Fig. 7:**
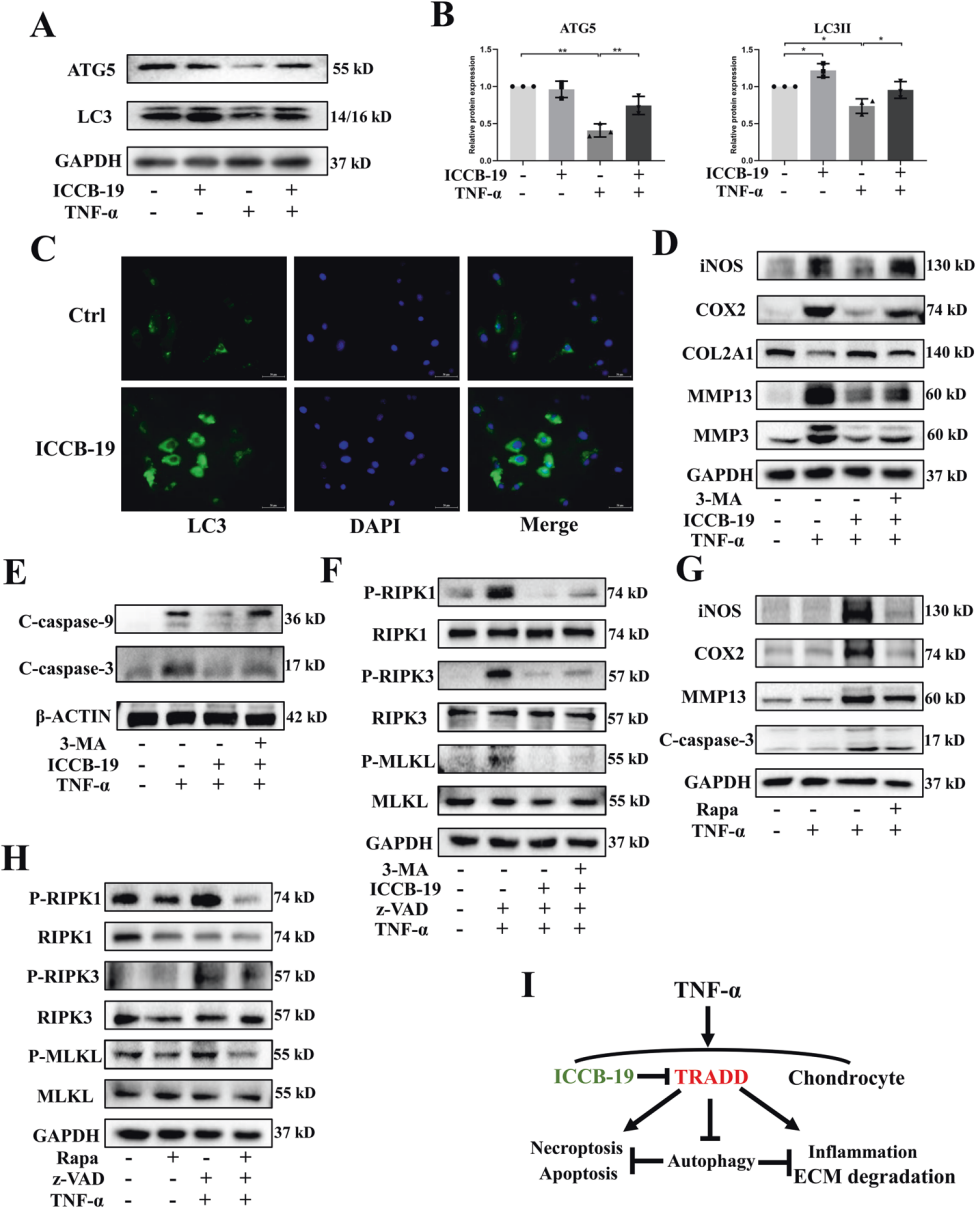
ICCB-19 protects against TNF-α-induced detrimental events via autophagy mechanism. After a pre-treatment of ICCB-19 for 2 h, chondrocytes were stimulated by TNF-α for 24 h. **A** the protein expression of ATG5 and LC3 was examined. **B** The semi-quantitative analysis of protein bands by image J. **C** Representative images of immunofluorescence staining of LC3 in chondrocytes, scale bar: 50 μm. **D**–**F** After a pre-treatment of ICCB-19 and 3-MA for 2 h, chondrocytes were stimulated by TNF-α for 24 h with or without z-VAD, the protein expression of iNOS, COX2, COL2A1, MMP13, MMP3, C-caspase-9, and C-caspase-3 were examined, and the phosphorylation of RIPK1, RIPK3, and MLKL as well as protein expression of these three markers were detected by western blot. **G**–**H** After a pre-treatment of Rapa for 2 h, chondrocytes were stimulated by TNF-α for 24 h with or without z-VAD, the protein expression of iNOS, COX2, MMP13, C-caspase-3 and the phosphorylation of RIPK1, RIPK3, and MLKL as well as protein expression of these three markers were detected by western blot. **I** The graphical scheme summarized the findings of protective role of ICCB-19 in TNF-α-caused detrimental events. Data are expressed as means ± SD. *p < 0.05, **p < 0.01. z-VAD, Z-VAD-FMK. C-caspase-9, cleaved-caspase-9. C-caspase-3, cleaved-caspase-3. P-, Phosphorylated-.

The above result indicated TRADD could modulate autophagic level of chondrocyte. Given that the important role of autophagy in maintaining cartilage homeostasis [19, 20]. We explored that whether ICCB-19 played the role in chondrocytes via autophagic mechanism. As expected, ICCB-19 treatment promoted autophagic process by promoting the expression of LC3, revealed by western blot and immunofluorescence (Fig. 7A–C). Besides, the ICCB-19 treatment also significantly rescued the downregulated expression of autophagy related 5 (ATG5) caused by TNF-α (Fig. 7A, B), restoring the level of autophagy in chondrocytes. To further verify that how autophagy is involved in the role of ICCB-19, we sought to regulate autophagic level of chondrocytes by using classical compounds. Firstly, 3-Methyladenine (3-MA) was primely added into culture medium to repress formation of autophagosome in chondrocytes. The effect of ICCB-19 and TNF-α with or without z-VAD were examined. The data showed that those effect of ICCB-19 against TNF-α with or without z-VAD were reversed by 3-MA treatment (Fig. 7D–F). Furthermore, to directly clarify the role of activated autophagy in those cellular events, a classical activator of autophagy, rapamycin (Rapa), was used. Rapa treatment inhibited protein expression of iNOS, COX2, MMP13, and cleaved-caspase-3 in chondrocytes under the stimulation of TNF-α (Fig. 7G). Moreover, Rapa also blocked the activation of RIPK1, RIPK3, and MLKL (Fig. 7H). Collectively, these results suggested that autophagy is indispensable to chondrocyte homeostasis and mediates protective role of ICCB-19 in TNF-α-caused detrimental events (Fig. 7I).

### ICCB-19 improves DMM-induced cartilage degeneration in vivo

**Fig. 8:**
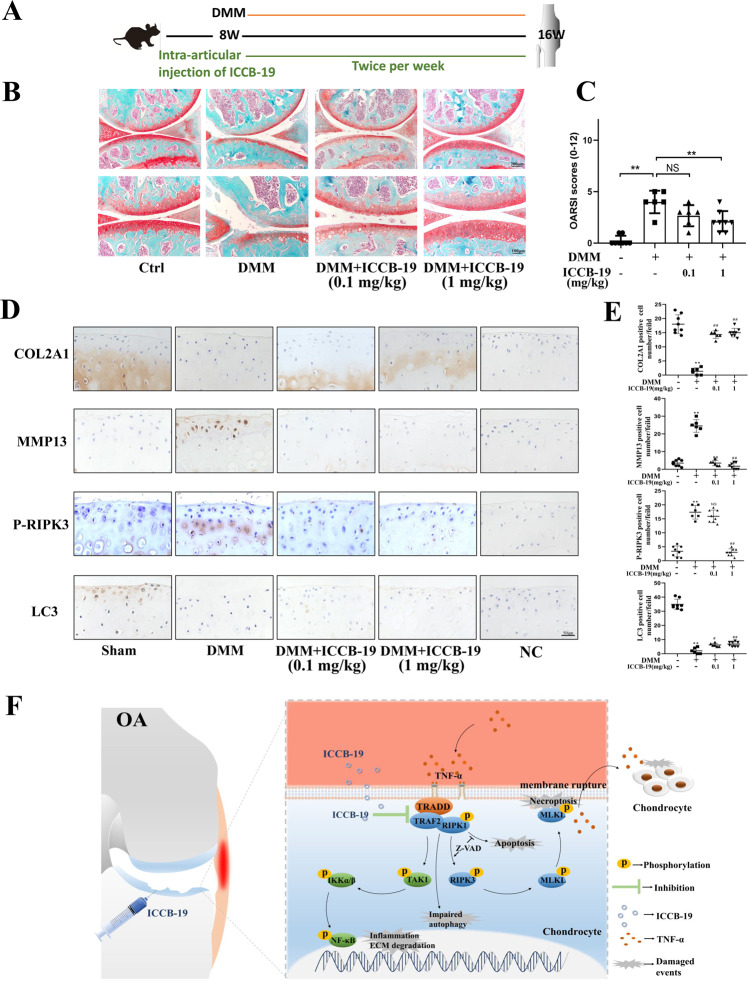
The role of ICCB-19 in DMM-induced cartilage degeneration. **A** Diagram of animal experiment design. **B** Representative images of Safranin O/Fast green staining of joint (scale bar: 200 or 100 μm). **C** OARSI scores for cartilage degeneration in four groups. **D** The protein expression of COL2A1, MMP13, P-RIPK3, and LC3 in cartilage were assayed by immunohistochemistry (scale bar: 50 μm), and quantitative analysis of positive cell number in field was performed (**E**). **F** Schematic representation of this study. The data in the figures represent the mean ± S.D. **P < 0.01 vs. the Sham group, ^#^*P* < 0.05 vs. the DMM group, ^##^*P* < 0.01 vs. the DMM group. NC negative control.

Based on the above finding, we further explore the therapeutic effects of ICCB-19 against OA progression. DMM surgery was performed to establish the post-traumatic OA model. To avoid possible system effect caused by ICCB-19, we chose a local administration of ICCB-19 by the intra-articular injection. A week after the surgery, ICCB-19 or PBS was injected into articular cavity twice a week in a 7-week period (Fig. 8A). DMM-surgery caused a moderate cartilage degeneration, shown by erosion or loss of cartilage matrix with an increased OARSI score compared to sham group. The administration of ICCB-19 (1 mg/kg) led to the significant attenuation of cartilage degeneration with a lower OARSI score (Fig. 8B–C). Consistently, increased positive staining of MMP13 and P-RIPK3 and reduced positive staining of COL2A1 and LC3 were found in articular cartilage of DMM-surgery mice. However, these changes of expression were significantly repressed by ICCB-19 treatment (Fig. 8D–E). These data demonstrated that the administration of ICCB-19 could attenuate cartilage degeneration of DMM mice (Fig. 8F).

## Discussion

To date, the pathogenesis of OA is not completely clear, and identifying therapeutic targets and agents for OA treatment is warranted. The current study establishes TRADD as a key node of TNF-α-related signals and associated cellular events in chondrocytes. We also demonstrate that the inhibitor of TRADD, ICCB-19, protects chondrocytes against TNF-α-induced damage and attenuates cartilage degeneration.

TNF-α, commonly found in synovial fluid from OA patients, contributes to the pathogenic processes of cartilage degeneration [6, 21]. This inflammatory mediator can cause inflammatory responses, break the ECM homeostasis, and lead to death of chondrocytes [7]. Therefore, TNF-α-related pathophysiology in OA is widely studied [22, 23]. Necroptosis is a type of regulated cell death and has been recently reported to be involved in OA development [24, 25]. Although in other studies, necroptosis is usually stimulated by TNF-α and a pan-caspase inhibitor, z-VAD-FMK [26], the pattern of the induction in chondrocytes is rarely reported. In this study, we sought to induce necroptosis of chondrocytes by combined treatment of TNF-α and z-VAD-FMK and successfully achieved it, evidenced by the activation of RIPK3 and MLKL. This finding will provide experimental condition of inducing chondrocyte necroptosis for relevant research. TNF-α-related cellular events are usually associated with its signal transduction and further a series of cascade reactions [27]. Therefore, a key upstream fortress of TNF-α signals will greatly affect the later cascades. TRADD has been considered as such a “tap”. *Trad* −*/−* mice show no apparent developmental defect [28], indicating that TRADD is dispensable during mouse development. Besides, *Trad* −*/−* mice showed less system inflammation compared to wild type mice [28, 29]. At cellular level, TRADD plays a dominating role in RIPK1 and NF-κB signaling pathway. TRADD deficiency considerably inhibited RIPK1-dependent extrinsic apoptosis [9] and NF-κB-dependent inflammation [29]. Consistent with that, we also found knocking down TRADD could inhibit TNF-α-induced inflammation and apoptosis. Additionally, TNF-α-caused ECM catabolism and decreased ECM anabolism is partially attenuated when TRADD is silenced. It is well reported that RIPK1 binds RIPK3 to initiate necroptosis in response to TNF stimulation. However, the study of Wang et al found that knockdown of RIPK1 failed to repress necroptosis, and double knockdown of TRADD and RIPK1 successfully inhibited necroptosis of L929 cells [30]. They further found that TRADD-RIPK3 is alternative pathway for necroptosis, evident by knockdown of RIPK1 facilitates the interaction between TRADD and RIPK3, leading to RIPK3-MLKL activation [30]. In this study, knockdown of TRADD not only inhibits the activation of RIPK1, and also inhibited necroptosis of chondrocyte, indicating TRADD might function on the upstream of RIPK1 and inhibition of TRADD might block RIPK1-dependent events and alternative pathway for necroptosis. Therefore, TRADD is considered as a pivotal hinge for TNF-α-related detrimental events and necroptosis of chondrocytes in this study.

We further investigated TRADD’s role in TNF-α signaling pathway, and demonstrated that TRADD could interaction with TRAF2 and mediate RIPK1-TAK1-NF-κB signal.

It has been well demonstrated that NF-κB is important for inflammation and ECM degradation in chondrocytes [31, 32], and the activation of RIPK1 contributes to apoptosis and necroptosis of chondrocytes [25, 33]. In addition, our group previously established the essential role of RIPK1 in interleukin 1 beta-induced activation of NF-κB and subsequent inflammatory response and ECM catabolism [16]. These evidence indicates RIPK1-NF-κB axis is closely related to chondrocyte damages and ECM degradation. In this study, we further explore TNF-α signals and highlight the important role of TRADD in RIPK1-TAK1-NF-κB signal and downstream cellular events in chondrocytes, suggesting that targeting TRADD may show a great therapeutic potential for OA.

Excitedly, the recent study by Yuan et al, demonstrated that ICCB-19 can specifically inhibit TRADD activity by binding to N-terminal domain of TRADD (TRADD-N), which interacts with the C-terminal domain and TRAF2 [9], partially inhibiting necroptosis of mouse embryonic fibroblasts. This finding inspired us to explore ICCB-19’ role in chondrocyte necroptosis and OA progression. In agreement with results of Yuan et al, we found that ICCB-19 treatment not only successfully inhibited the downstream signaling of TRADD, but also alleviated TNF-α-induced necroptosis of chondrocytes. Notably, this compound also exerted its chondroprotective effect by attenuating inflammatory responses, ECM degradation, and apoptosis. It is clearly demonstrated that the phosphorylated MLKL moves from the cytosol to the plasma and intracellular membranes, where it directly disrupts membrane integrity followed by the uncontrollable release of intracellular material including inflammatory mediators, resulting in necrotic death [34, 35]. Based on these characteristics of necroptosis, we performed indirect co-culture experiment and confirmed release of intracellular substrates in TNF-α + z-VAD-induced chondrocytes, which further promoted necroptotic process. This phenomenon was also attenuated by ICCB-19. These findings support the hypothesis that modulation of TRADD by ICCB-19 could mitigate necroptosis and OA-like phenotypes of chondrocytes.

Autophagy is an endogenous process necessary for the turnover of organelles and maintaining chondrocyte homeostasis [36]. Autophagy is impaired during OA development and involved in multiple pathologic processes. It has been reported that inhibition of autophagy in normal cartilage could trigger cartilage degeneration [37], and on the contrary, activation of autophagy could inhibit cartilage degeneration and apoptosis [38–40]. Consistently, our data showed that TNF-α also impaired autophagy while the activation of autophagy by rapamycin could counteract TNF-α-caused changes of phenotypes. The previous study has demonstrated that autophagy protein ATG16L1 prevents necroptosis in the intestinal epithelium [41]. Tetsuji Miura et al. have reported that mTORC1 inhibition promotes autophagy and protects cardiomyocytes from necroptosis by a TFEB-dependent mechanism [42]. However, the regulatory role of autophagy in necroptosis is not clear in chondrocyte. One important observation of this study is that the anti-necroptotic function of ICCB-19 in chondrocytes is related to its regulation of autophagy. Activation of autophagy by rapamycin could protect against TNF-α + z-VAD-caused necroptotic phenotypes while inhibition of autophagy by 3-MA heavily reversed effects of ICCB-19 on necroptosis and ECM degradation, indicating that autophagy could also regulate necroptotic process in chondrocytes. Taken together, these findings confirm the essential role of autophagy mediating chondroprotective function of ICCB-19.

Based on the protective effects of ICCB-19 on chondrocytes, we further evaluated anti-osteoarthritic effects of this agent in vivo. It is noted that ICCB-19 is easily dissolved in saline, which makes it available for in vivo experiment and future clinical application. To avoid systemic inhibition of TRADD and possible side effects, we performed the intra-articular injection of ICCB-19 and found this agent was able to halt cartilage degeneration in a DMM-induced model of OA. Meanwhile, ICCB-19 supplementation inhibited activation of RIPK3 and promoted LC3 expression in articular cartilage. This study firstly uncovers anti-osteoarthritic effects of ICCB-19 on pre-clinical experiment and indicates that ICCB-19 might be potential agent for clinical treatment of OA.

The current study has several limitations. The regulatory mechanism of TRADD expression or activity in chondrocyte is still unknown. How TRAF2 interacts TRADD and mediates the function of TRADD in chondrocytes remains unclear. In addition, to confirm TRADD’ role in vivo, an inducible cartilage-specific genetic model is needed and the effect of TRADD overexpression on OA development should be investigated. We will continue to study these contents deeply in the future. Apart from mediating inflammation pathway, TRADD is also involved in the regulation of autophagy [9]. Inhibition of TRADD can activate autophagy to maintain cellular homeostasis [9]. In our results, ICCB-19 treatment could promote autophagic level of chondrocyte even without TNF-α exposure (Fig. 7). It has been reported that increased autophagic level could attenuate age-related diseases including OA [37, 43, 44]. Therefore, it is significant to further explore the therapeutic role of ICCB-19 in the model of age-related OA. These issues were expected to be addressed in the future.

## Conclusion

In summary, our study provides novel information on essential role of TRADD in TNF-α-related signals and OA-like phenotypes in chondrocytes, and identifies ICCB-19 as a key regulator of TNF-α-TRADD axis for cartilage degeneration. Besides, autophagy is indispensable for protective role of ICCB-19. Translationally, local administration of ICCB-19 alleviates cartilage degeneration in the DMM-induced OA model, suggesting that targeting TRADD by ICCB-19 is a promising therapeutic strategy for OA.

## Materials and methods

### Human samples

OA cartilage was obtained from patients with OA after total knee replacement surgery at the Tongji Hospital (*n* = 10). Control cartilage was collected from four amputees without a history of OA. The collection of human cartilage samples was approved by the Ethics Committee of Tongji Hospital (TJ-IRB20210905). Informed consent was obtained from all participants.

### Reagent

ICCB-19 Hydrochloride (S6078, 99.25% purity) and apoptosis inhibitor z-VAD-FMK (S7023, 99.25% purity) were purchased from Selleck Chemicals. ICCB-19 was dissolved in PBS and z-VAD-FMK was dissolved in dimethyl sulfoxide. Recombinant mouse TNF-α (410-MT) was purchased from the R&D system (Minneapolis, Minnesota, USA) and dissolved in PBS containing 0.5% bovine serum albumin. Fetal bovine serum (FBS) and Dulbecco modified Eagle’s Culture Medium F12 (DMEM/F12) were provided by Gibco (New York, USA). Antibodies to iNOS (#sc-7271), COX2 (#12882), P-RIPK1 (Ser166, #44590), P-RIPK3 (Thr231/Ser232, #91702), TAK1 (#5206), P-TAK1 (Ser412, #9339), IκBα (#4812), P-IκBα (Ser32, #2859), P-P65 (Ser536, #3033), P-IKK α/β (Ser176/180, # 2697), IKK β (#8943), and LC3B (#2775) were purchased from Cell Signaling Technology Inc (Beverly, USA). Antibodies to MLKL (ab243142), P-MLKL (Ser345, ab196436), TRADD (ab110644), RIPK1 (ab300617), and MMP13 (#ab39012) were purchased from Abcam (Cambridge, UK). Antibodies to COL2A1 (15943-1-AP), P65 (#10745-1-AP), RIPK3 (#17563-1-AP), cleaved-caspase-3 (#19677-1-AP), and ATG5 (#10181-2-AP) were purchased from Proteintech Group (Wuhan, China). Cleaved-caspase-9 (#A0281), P-RIPK3 (used for immunohistochemistry) were provided by ABclonal (Wuhan, China), Antibodies to β-ACTIN (#BM0627), GAPDH (#BM3876), MMP3 (#BM4074), FITC-conjugate goat anti-rabbit secondary antibody (#BA1105), trypsin, and type II collagenase were purchased from Boster (Wuhan, China).

### Isolation and culture of mouse chondrocytes

Chondrocytes were isolated from 5-day-old C57BL/6 mice (provided by The Experimental Animal Center of Tongji Hospital). Breifly, cartilage was removed from the knee joint and chopped, then treated with 0.25% trypsin at 37 °C for 30 min. After trypsin removal, cartilage tissue was treated with 0.25% type II collagenase solution at 37 °C for 6 h. Cells were then collected and cultured in DMEM/F12 medium containing 10% FBS, and passed at 80% confluence. The 1st and 2nd passage chondrocytes in culture plates are used for our experiments.

### Measurement of cell viability

According to the instruction, the effect of ICCB-19 on chondrocyte viability was determined by cell counting kit 8 assay (CCK-8, Boster, China). In brief, chondrocytes (5000–10000 cells/well) were seeded on 96-well plate, allowed to adhere for 24 h, and then treated with different concentrations of ICCB-19 for 24 h. Subsequently, 10 μl CCK-8 solution was added to each well and the cells were cultured in darkness for 1 h. Finally, the absorbance of the wells was measured at 450 nm using a microplate reader (molecular facility in Sunnyvale, California).

### Toluidine blue staining

Toluidine blue staining was used to observe the morphology of chondrocytes according to the manufacturer’s instruction. The chondrocytes were washed with PBS and fixed with 4% paraformaldehyde for 20 min at room temperature. The cells were stained with toluidine blue solution for 15 min and then excessive dye was removed. Afterwards, the cells were washed with PBS three times and observed using a fluorescence microscope (Evosfl auto, Life Technologies, United States).

### Co-immunoprecipitation (Co-IP)

The extracted proteins were incubated with 2 μg of TRADD antibody or Rabbit IgG (B900610, Proteintech) overnight at 4 °C. Next, 50 μl of Protein A/G Magnetic Beads (HY-K0202, MCE) was added to each incubation sample for 2 h at 4 °C. The beads were washed five times with 1 × PBS and the coimmunoprecipitated proteins were lysed by 80 μl of lysis solution. The lysis was eluted by 20 μl of 5 × SDS buffer and heated at 95 °C for 10 min. The interaction between proteins was examined using western blot.

### Western blot

The cells were washed twice with cold PBS and treated on ice with RIPA lysis buffer and 1% protease inhibitor mixture for 30 min, followed by centrifugation at 12800 g for 30 min. Protein concentrations were measured using the bicinchoninic acid assay kit kit (Boster, China). The protein sample (25 μg) was then separated on 12% SDS-Page gel and transferred to PVDF membrane (Millipore, Billerica, MA). The membrane was sealed with 5% bovine serum albumin dissolved in Tris buffered saline Tween 20 (TBST) for 1 h and then incubated overnight with primary antibodies (1:1000-1:300) at 4 °C. Then the membrane was cleaned 3 times in TBST and incubated with horseradish peroxidase-conjugated secondary antibody (1:5000) for 1 h. The protein bands were visualized using an electrochemiluminescent substrate Kit (Boster, China) and analyzed using a Bio-RAD scanner (Bio-RAD, Hercules, CA).

### Annexin V-FITC/PI staining

Annexin V-FITC/PI kit (Yeasen, China) was used to detect apoptotic cells. After treatment, the chondrocytes were collected and washed twice with cold PBS, followed by staining with Annexin V-FITC/PI in the dark. After 15 min, cells were analyzed using FACS Calibur flow cytometry (BD Biosciences, USA).

### Immunofluorescence

Chondrocytes were seeded on 24-well plates for LC3 staining. After the treatment, the cells were fixed with 4% paraformaldehyde at room temperature for 10 min. After fixed and washed with PBS for 3 times, the cells were permeabilized with PBS containing 0.1% Triton X-100 for 10 min and blocked with 5% BSA for 1 h. Then, chondrocytes were incubated with anti-LC3 primary antibody overnight at 4 °C. After washing with PBS for 3 times, cells were incubated with FITC-conjugated goat anti-rabbit secondary antibody in darkness for 1 h. Finally, the cells were washed with PBS for 3 times and stained with DAPI for 10 min. Images were obtained using a fluorescence microscope (Evosfl auto, Life Technologies, United States).

### TUNEL staining

TUNEL staining was used to detect the degree of DNA damage that represents apoptosis level. After the treatment, the chondrocytes were fixed and stained with a TUNEL Apoptosis Detection Kit (Alexa Fluor 488, Yeasen, China) according to the manufacturer’s protocol, and the nuclei were stained with DAPI. Slices were captured under a fluorescence microscope (Evosfl auto, Life Technologies, United States).

### Small interfering RNA (siRNA), plasmids, and transfection

Chondrocytes were transfected with specific TRADD siRNA synthesized by RiboBio, Guangzhou, China. The sequence of TRADD siRNA is as follows: sense strand 5 ‘-CGACAGACCCTCCATCTA-3’. The cells were cultured in six-well plates. When cell density reached 60–70%, negative control (100 nM) or TRADD siRNA (100 nM) was transfected with lipofectamine 3000 transfection reagent (Thermo Fisher, UT, USA) for 48 h according to the manufacture’s instruction. Subsequently, the cells were cultured in fresh DMEM/F12 medium for further treatment. The pcDNA3.1 plasmid of *Tradd* and negative control plasmid were synthesized by Wuhan AuGCT DNA-SYN Biotechnology. The construct was confirmed by DNA sequencing. When reaching 60–70% of confluence, the cells were transfected with 1 μg purified plasmid using Lipofectamine 3000.

### Animal experiments procedure

Thirty-two 8-week-old male C57BL/6 mice with body weight of (25 g ± 3 g) were provided by Experimental Animal Center of Tongji Medical College. The mice were placed in a clean, ventilated room and given a standard diet and normal light and dark cycles. All animal experiments have been approved by the Animal Care and Use Committee of Tongji Medical College (IACUC Number:2757). Thirty-two mice were randomly divided into sham operation group, DMM group, DMM + ICCB-19 (0.1 mg/kg) and DMM + ICCB-19 (1 mg/kg) groups, with 8 mice in each group. DMM is a standard surgical approach for establishing the model of experimental OA. Mice were anesthetized by intraperitoneal injection of 2% pentobarbital (40 mg/kg) and the surgery was performed on the right knee of mice according to published protocol [45]. A week after the surgery, mice in DMM + ICCB-19 group were intra-articularly injected with ICCB-19 twice a week for consecutive seven weeks. ICCB-19 was dissolved in 10 µl solution of normal saline. The sham and DMM groups received only 10 µl normal saline. Eight weeks after the surgery, joint tissue from each right knee was collected for further analysis.

### Histological examination

After euthanasia, all specimens were fixed in 4% paraformaldehyde for 48 h, decalcified with 10% EDTA solution for 25 days, and then, embedded in paraffin. The knee joint was cut from the medial joint space to a 5 µm thick sagittal plane using a microscopic cutter (Leica Biossystems, USA). Subsequently, sections were stained with safranin O/fast green. The Osteoarthritis Research Society International (OARSI) histopathological scoring system was used to assess the severity of cartilage degeneration [46]. The scoring was done by two independent investigators, who were blind to grouping. Immunohistochemistry was performed as described previously [47]. The sections were incubated with anti-LC3, MMP13, P-RIPK3, and COL2A1 antibodies to observe expression changes of these markers.

### Statistical analysis

Graph Pad Prism 8.0 software was used for statistical analysis. Results were revealed as mean ± standard deviation (SD). Student *T* test (unpaired, two-tailed) was used for statistical analysis between the two groups. Comparisons between more than two groups were performed by ANOVA and Tukey test. *P* values <0.05 was statistically significant. *N* = 3 means the in vitro experiments were performed at least three biological replicates.

## Supplementary information

Original Data File

## Data Availability

Full images of western blotting had been included in Supplementary Materials. Other data are available from the corresponding authors for reasonable grounds.

## References

[1] Hunter DJ, Bierma-Zeinstra S (2019). Osteoarthritis. Lancet.

[2] Mobasheri A, Batt M (2016). An update on the pathophysiology of osteoarthritis. Ann Phys Rehabil Med.

[3] Silverwood V, Blagojevic-Bucknall M, Jinks C, Jordan JL, Protheroe J, Jordan KP (2015). Current evidence on risk factors for knee osteoarthritis in older adults: a systematic review and meta-analysis. Osteoarthr Cartil.

[4] Guilak F, Nims RJ, Dicks A, Wu C, Meulenbelt I (2018). Osteoarthritis as a disease of the cartilage pericellular matrix. Matrix Biol.

[5] Pitsillides AA, Beier F (2011). Cartilage biology in osteoarthritis—lessons from developmental biology. Nat Rev Rheumatol.

[6] Kapoor M, Martel-Pelletier J, Lajeunesse D, Pelletier J, Fahmi H (2011). Role of proinflammatory cytokines in the pathophysiology of osteoarthritis. Nat Rev Rheumatol.

[7] Ma C, Wu C, Jou I, Tu Y, Hung C, Hsieh P (2018). PKR activation causes inflammation and MMP-13 secretion in human degenerated articular chondrocytes. Redox Biol.

[8] Hsu H, Xiong J, Goeddel DV. The TNF receptor 1-associated protein TRADD signals cell death and NF-kappa B activation. In, 1995:495-504. 10.1016/0092-8674(95)90070-5 7758105

[9] Xu D, Zhao H, Jin M, Zhu H, Shan B, Geng J (2020). Modulating TRADD to restore cellular homeostasis and inhibit apoptosis. Nature.

[10] Pobezinskaya YL, Liu Z (2014). The role of TRADD in death receptor signaling. Cell Cycle.

[11] Wajant H, Scheurich P (2011). TNFR1-induced activation of the classical NF-κB pathway. FEBS J.

[12] Ea C, Deng L, Xia Z, Pineda G, Chen ZJ (2006). Activation of IKK by TNFalpha requires site-specific ubiquitination of RIP1 and polyubiquitin binding by NEMO. Mol Cell.

[13] Zhang D, Shao J, Lin J, Zhang N, Lu B, Lin S (2009). RIP3, an energy metabolism regulator that switches TNF-induced cell death from apoptosis to necrosis. Science..

[14] Yamazaki K, Gohda J, Kanayama A, Miyamoto Y, Sakurai H, Yamamoto M (2009). Two mechanistically and temporally distinct NF-κB activation pathways in IL-1 signaling. Sci Signal.

[15] Liu WJ, Ye L, Huang WF, Guo LJ, Xu ZG, Wu HL (2016). p62 links the autophagy pathway and the ubiqutin–proteasome system upon ubiquitinated protein degradation. Cell Mol Biol Lett.

[16] Liang S, Wang Z, Zhang Z, Chen K, Lv Z, Wang Y (2019). Decreased RIPK1 expression in chondrocytes alleviates osteoarthritis via the TRIF/MyD88-RIPK1-TRAF2 negative feedback loop. Aging.

[17] Gaud G, Guillemot D, Jacob Y, Favre M, Vuillier F (2013). EVER2 protein binds TRADD to promote TNF-α-induced apoptosis. Cell Death Dis.

[18] Pasparakis M, Vandenabeele P (2015). Necroptosis and its role in inflammation. Nature.

[19] Lotz MK, Caramés B (2011). Autophagy and cartilage homeostasis mechanisms in joint health, aging and OA. Nat Rev Rheumatol.

[20] Sun K, Jing X, Guo J, Yao X, Guo F (2021). Mitophagy in degenerative joint diseases. Autophagy.

[21] Boffa A, Merli G, Andriolo L, Lattermann C, Salzmann GM, Filardo G (2021). Synovial fluid biomarkers in knee osteoarthritis: a systematic review and quantitative evaluation using BIPEDs criteria. Cartilage.

[22] Lee SW, Rho JH, Lee SY, Kim JH, Cheong JH, Kim HY (2015). Leptin protects rat articular chondrocytes from cytotoxicity induced by TNF-alpha in the presence of cyclohexamide. Osteoarthr Cartil.

[23] Sun K, Luo J, Jing X, Guo J, Yao X, Hao X (2019). Astaxanthin protects against osteoarthritis via Nrf2: a guardian of cartilage homeostasis. Aging.

[24] Riegger J, Brenner RE (2019). Evidence of necroptosis in osteoarthritic disease: investigation of blunt mechanical impact as possible trigger in regulated necrosis. Cell Death Dis.

[25] Zhang C, Lin S, Li T, Jiang Y, Huang Z, Wen J (2017). Mechanical force-mediated pathological cartilage thinning is regulated by necroptosis and apoptosis. Osteoarthr Cartil.

[26] Ogasawara M, Yano T, Tanno M, Abe K, Ishikawa S, Miki T (2017). Suppression of autophagic flux contributes to cardiomyocyte death by activation of necroptotic pathways. J Mol Cell Cardiol.

[27] Horiuchi T, Mitoma H, Harashima SI, Tsukamoto H, Shimoda T (2010). Transmembrane TNF-: structure, function and interaction with anti-TNF agents. Rheumatology.

[28] Dowling JP, Alsabbagh M, Del Casale C, Liu Z, Zhang J (2019). TRADD regulates perinatal development and adulthood survival in mice lacking RIPK1 and RIPK3. Nat Commun.

[29] Ermolaeva MA, Michallet M, Papadopoulou N, Utermöhlen O, Kranidioti K, Kollias G (2008). Function of TRADD in tumor necrosis factor receptor 1 signaling and in TRIF-dependent inflammatory responses. Nat Immunol.

[30] Wang L, Chang X, Feng J, Yu J, Chen G (2020). TRADD Mediates RIPK1-Independent Necroptosis Induced by Tumor Necrosis Factor. Front Cell Developmental Biol.

[31] Sueishi T, Akasaki Y, Goto N, Kurakazu I, Toya M, Kuwahara M (2020). GRK 5 inhibition attenuates cartilage degradation via decreasedNF ‐κB signaling. Arthritis Rheumatol.

[32] Yan H, Duan X, Pan H, Holguin N, Rai MF, Akk A (2016). Suppression of NF-κB activity via nanoparticle-based siRNA delivery alters early cartilage responses to injury. Proc Natl Acad Sci.

[33] Qiu X, Zhuang M, Lu Z, Liu Z, Cheng D, Zhu C (2019). RIPK1 suppresses apoptosis mediated by TNF and caspase-3 in intervertebral discs. J Transl Med.

[34] Wang H, Sun L, Su L, Rizo J, Liu L, Wang L (2014). Mixed lineage kinase domain-like protein MLKL causes necrotic membrane disruption upon phosphorylation by RIP3. Mol Cell.

[35] Bertheloot D, Latz E, Franklin BS (2021). Necroptosis, pyroptosis and apoptosis: an intricate game of cell death. Cell Mol Immunol.

[36] Rockel JS, Kapoor M (2016). Autophagy: controlling cell fate in rheumatic diseases. Nat Rev Rheumatol.

[37] Bouderlique T, Vuppalapati KK, Newton PT, Li L, Barenius B, Chagin AS (2016). Targeted deletion of Atg5 in chondrocytes promotes age-related osteoarthritis. Ann Rheum Dis.

[38] Zhang Y, Vasheghani F, Li Y, Blati M, Simeone K, Fahmi H (2015). Cartilage-specific deletion of mTOR upregulates autophagy and protects mice from osteoarthritis. Ann Rheum Dis.

[39] Caramés B, Hasegawa A, Taniguchi N, Miyaki S, Blanco FJ, Lotz M (2012). Autophagy activation by rapamycin reduces severity of experimental osteoarthritis. Ann Rheum Dis.

[40] Li J, Jiang M, Yu Z, Xiong C, Pan J, Cai Z (2022). Artemisinin relieves osteoarthritis by activating mitochondrial autophagy through reducing TNFSF11 expression and inhibiting PI3K/AKT/mTOR signaling in cartilage. Cell Mol Biol Lett.

[41] Matsuzawa-Ishimoto Y, Shono Y, Gomez LE, Hubbard-Lucey VM, Cammer M, Neil J (2017). Autophagy protein ATG16L1 prevents necroptosis in the intestinal epithelium. J Exp Med.

[42] Abe K, Yano T, Tanno M, Miki T, Kuno A, Sato T (2019). mTORC1 inhibition attenuates necroptosis through RIP1 inhibition-mediated TFEB activation. Biochimica et Biophysica Acta (BBA) - Mol Basis Dis.

[43] Leidal AM, Levine B, Debnath J (2018). Autophagy and the cell biology of age-related disease. Nat Cell Biol.

[44] Meckes JK, Caramés B, Olmer M, Kiosses WB, Grogan SP, Lotz MK (2017). Compromised autophagy precedes meniscus degeneration and cartilage damage in mice. Osteoarthr Cartil.

[45] Ma HL, Blanchet TJ, Peluso D, Hopkins B, Morris EA, Glasson SS (2007). Osteoarthritis severity is sex dependent in a surgical mouse model. Osteoarthr Cartil.

[46] Schmitz N, Laverty S, Kraus VB, Aigner T (2010). Basic methods in histopathology of joint tissues. Osteoarthr Cartil.

[47] Sun K, Luo J, Jing X, Xiang W, Guo J, Yao X (2021). Hyperoside ameliorates the progression of osteoarthritis: An in vitro and in vivo study. Phytomedicine.

