# Supplementary material for: Inhibition of TRADD ameliorates chondrocyte necroptosis and osteoarthritis by blocking RIPK1-TAK1 pathway and restoring autophagy
Source: Cell Death Discov. 2023 Mar 31;9:109. doi: 10.1038/s41420-023-01406-0 (PMC10066284; doi:10.1038/s41420-023-01406-0)
Supplement: Supplementary file 1 — Original Data File [file 41420_2023_1406_MOESM1_ESM.pdf]

Fig.1

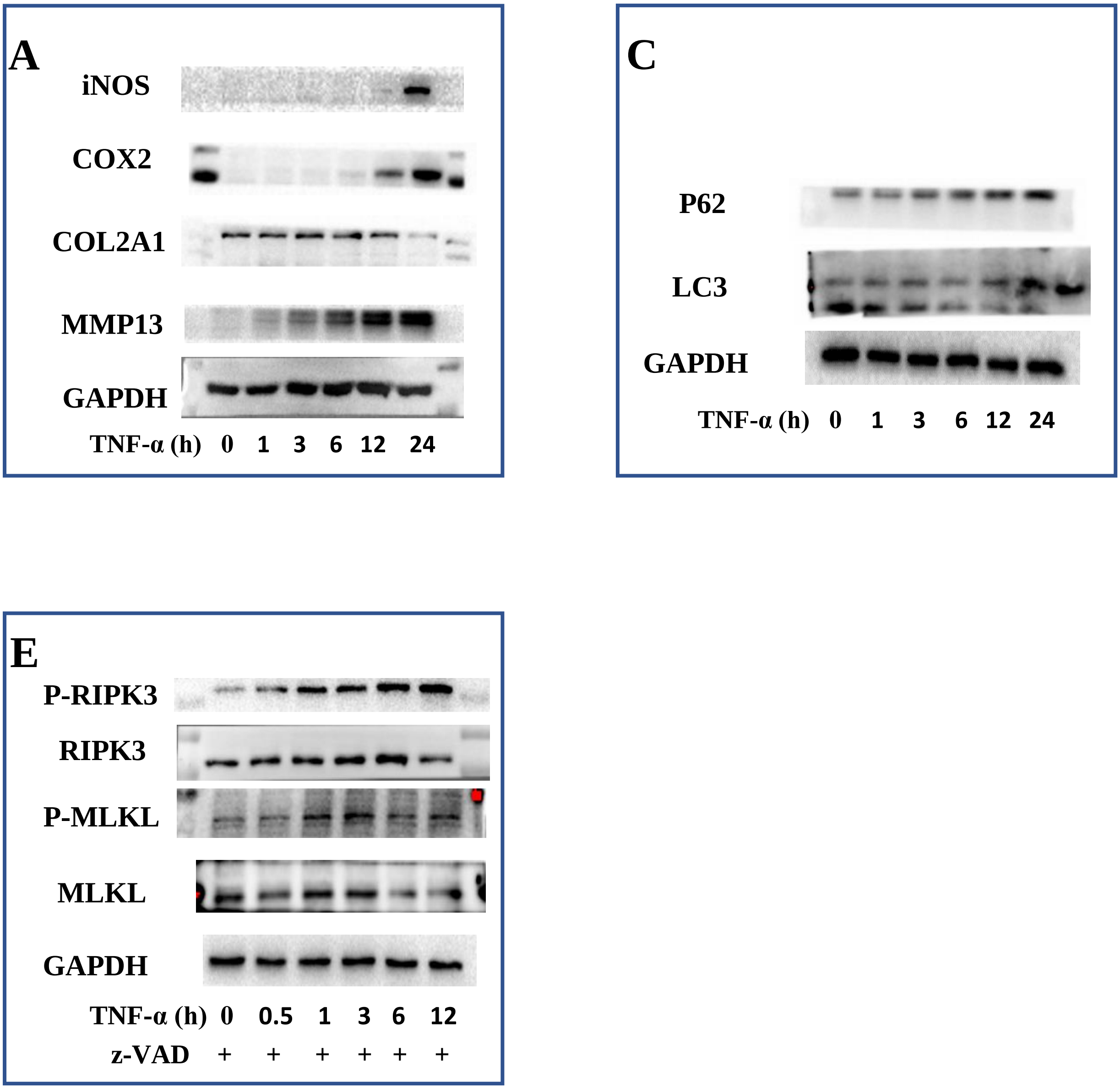
A
iNOS
COX2
COL2A1
MMP13
GAPDH
TNF-α (h) 0 1 3 6 12 24
C
P62
LC3
GAPDH
TNF-α (h) 0 1 3 6 12 24
E
P-RIPK3
RIPK3
P-MLKL
MLKL
GAPDH
TNF-α (h) 0 0.5 1 3 6 12
z-VAD + + + + + +

Fig.2

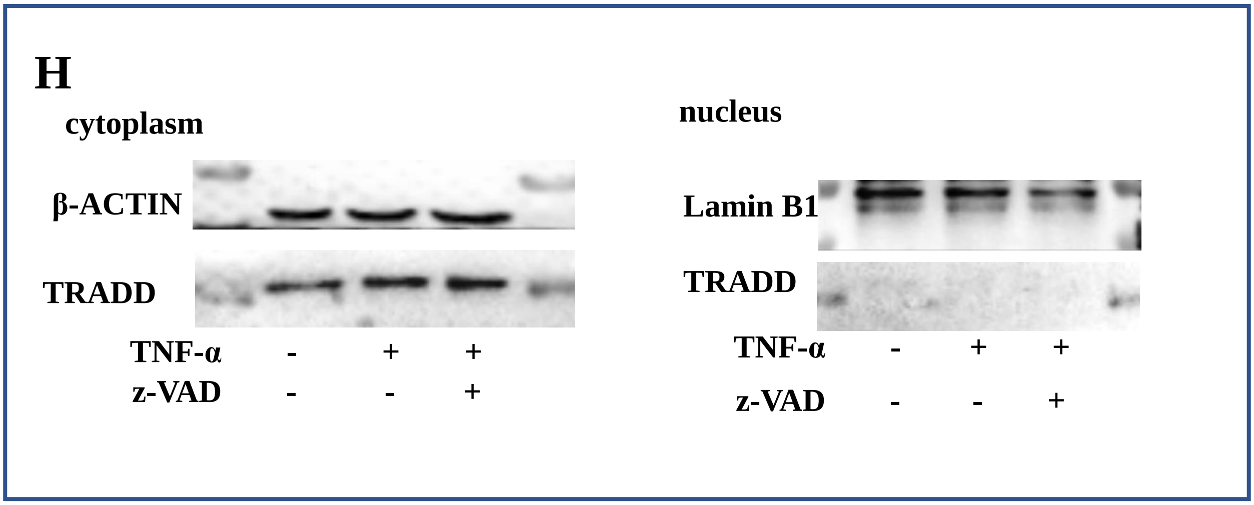

H
cytoplasm
nucleus
β-ACTIN
Lamin B1
TRADD
TRADD
TNF-α - + +
z-VAD - - +
TNF-α - + +
z-VAD - - +

Fig.3

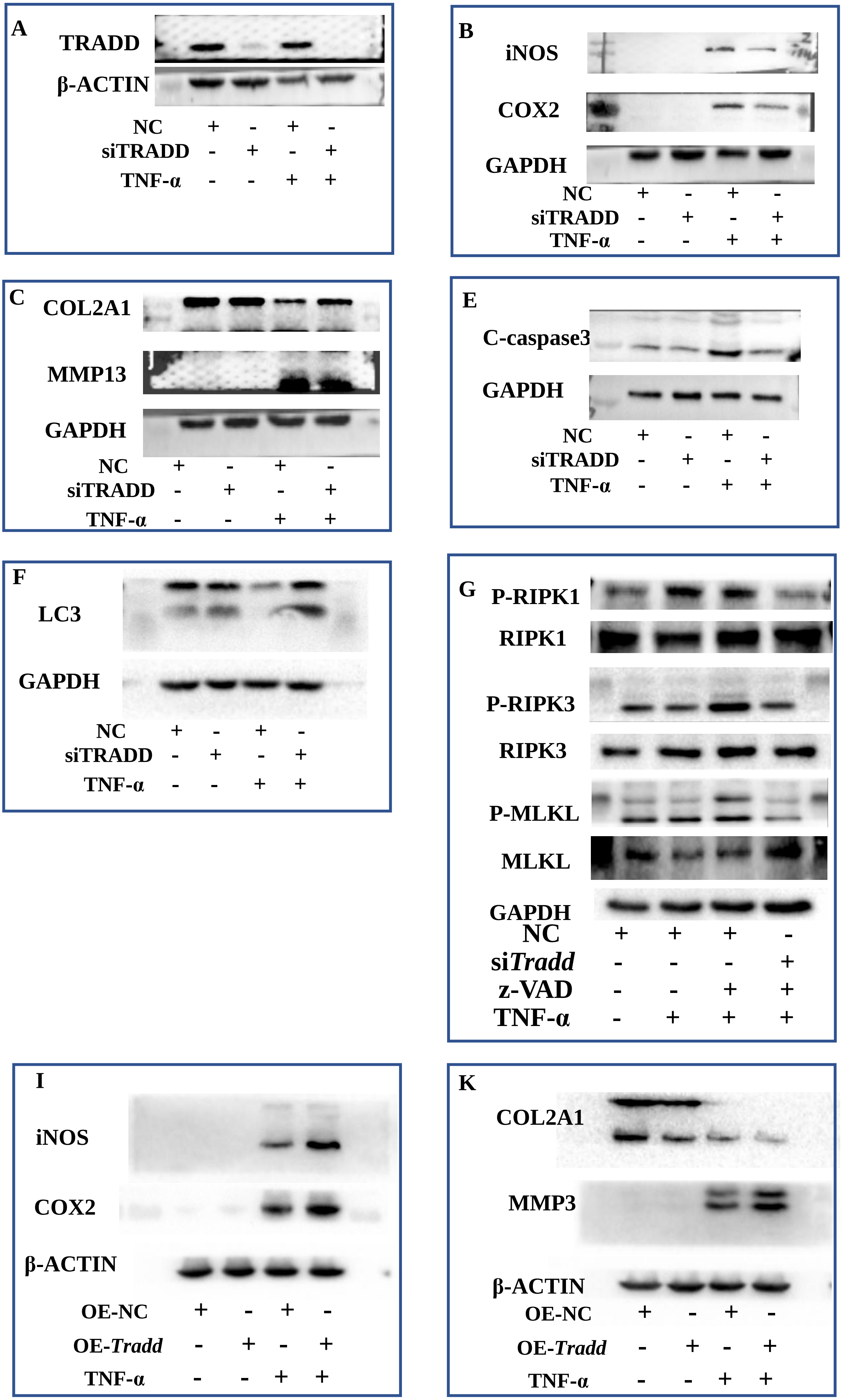

A
TRADD
β-ACTIN
NC + - + -
siTRADD - + - +
TNF-α - - + +
B
iNOS
COX2
GAPDH
NC + - + -
siTRADD - + - +
TNF-α - - + +
C
COL2A1
MMP13
GAPDH
NC + - + -
siTRADD - + - +
TNF-α - - + +
E
C-caspase3
GAPDH
NC + - + -
siTRADD - + - +
TNF-α - - + +
F
LC3
GAPDH
NC + - + -
siTRADD - + - +
TNF-α - - + +
G
P-RIPK1
RIPK1
P-RIPK3
RIPK3
P-MLKL
MLKL
GAPDH
NC + + + -
siTradd - - - +
z-VAD - - + +
TNF-α - + + +
I
iNOS
COX2
β-ACTIN
OE-NC + - + -
OE-Tradd - + - +
TNF-α - - + +
K
COL2A1
MMP3
β-ACTIN
OE-NC + - + -
OE-Tradd - + - +
TNF-α - - + +

Fig.4

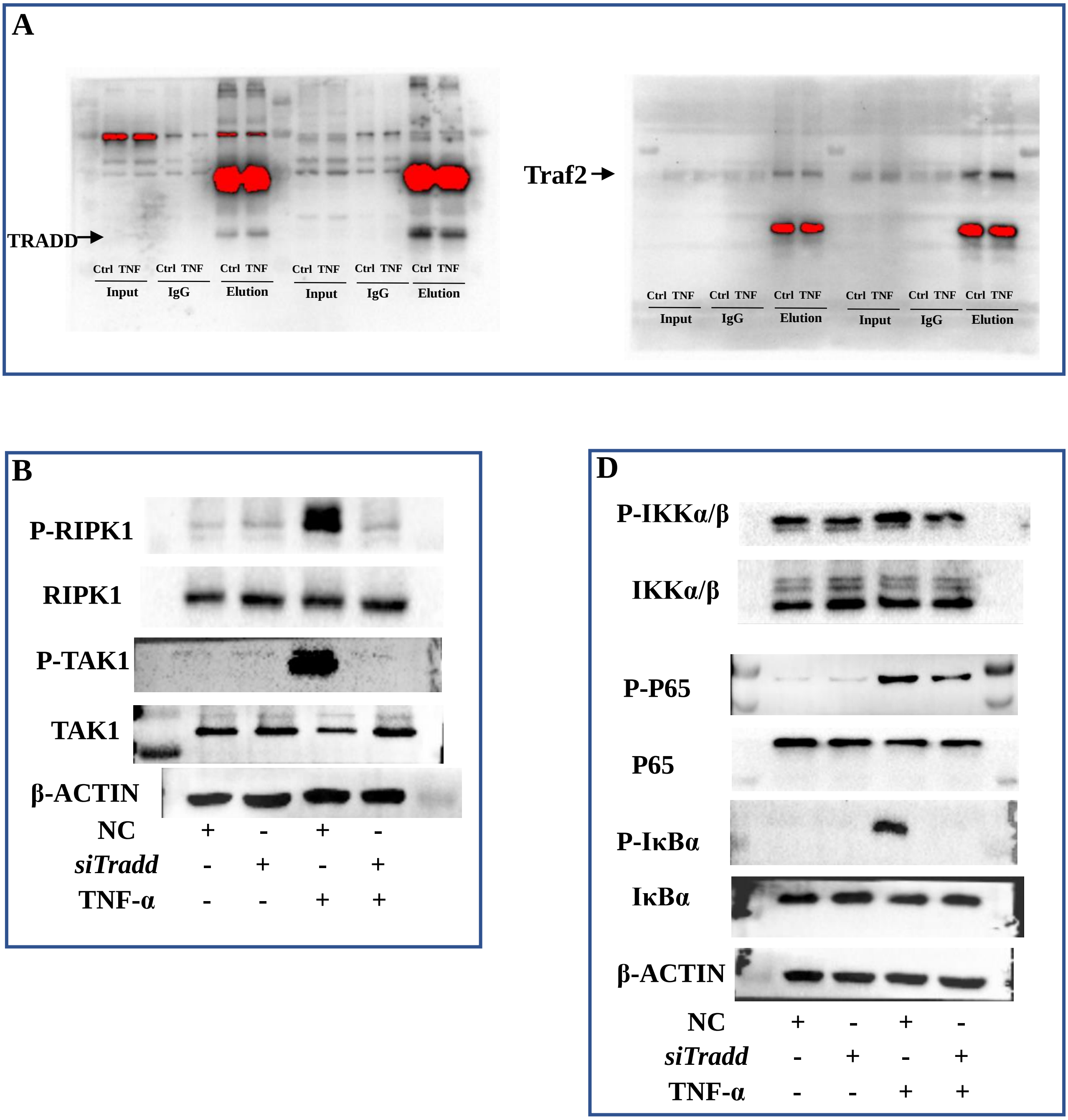

A
TRADD
Ctrl TNF
Input
IgG
Elution
Traf2
B
P-RIPK1
RIPK1
P-TAK1
TAK1
β-ACTIN
NC + - + -
siTradd - + - +
TNF-α - - + +
D
P-IKKα/β
IKKα/β
P-P65
P65
P-IκBα
IκBα
β-ACTIN
NC + - + -
siTradd - + - +
TNF-α - - + +

Fig.5

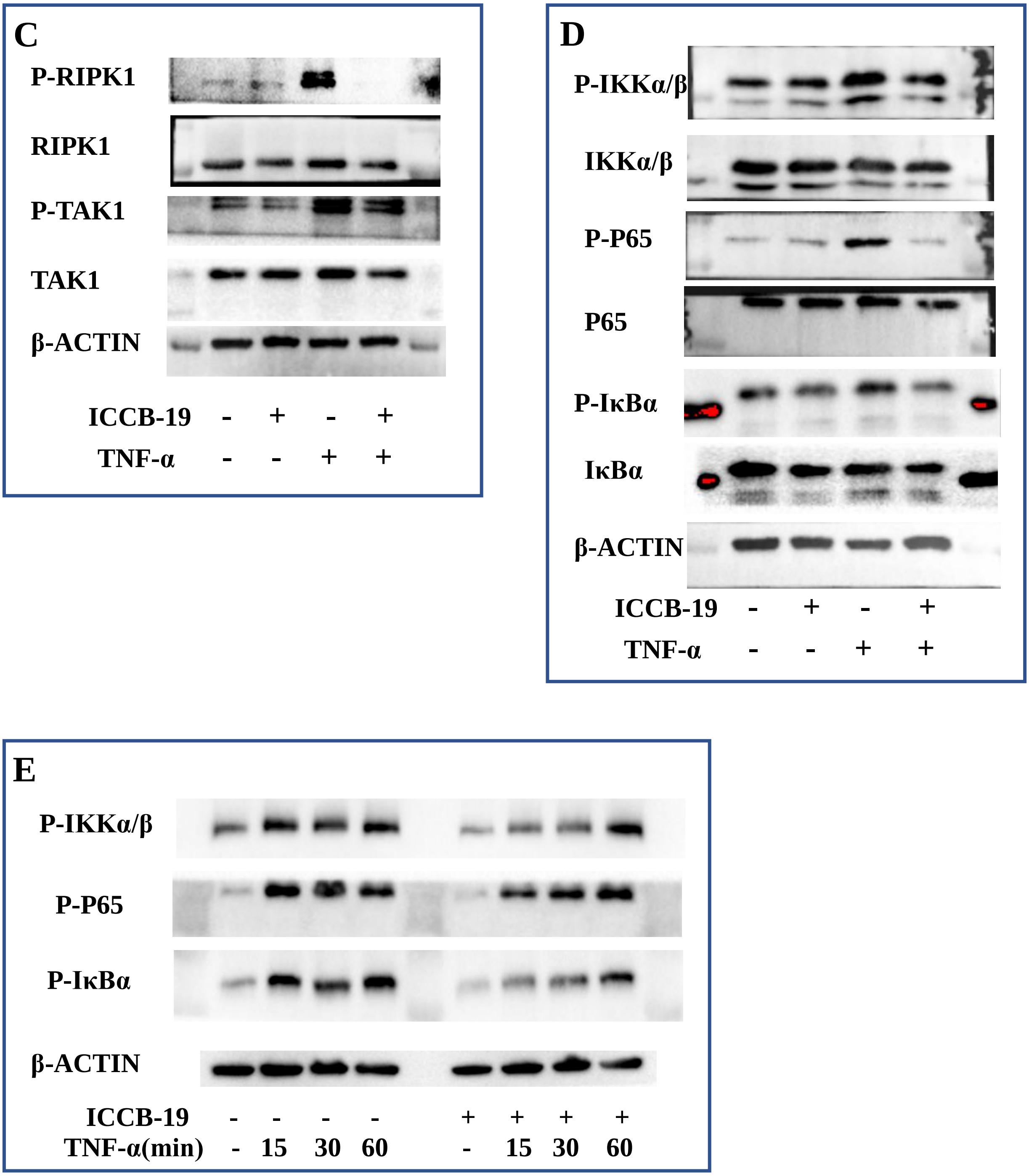
C
P-RIPK1
RIPK1
P-TAK1
TAK1
β-ACTIN
ICCB-19 - + - +
TNF-α - - + +
D
P-IKKα/β
IKKα/β
P-P65
P65
P-IκBα
IκBα
β-ACTIN
ICCB-19 - + - +
TNF-α - - + +
E
P-IKKα/β
P-P65
P-IκBα
β-ACTIN
ICCB-19 - - - - + + + +
TNF-α(min) - 15 30 60 - 15 30 60

Fig.6

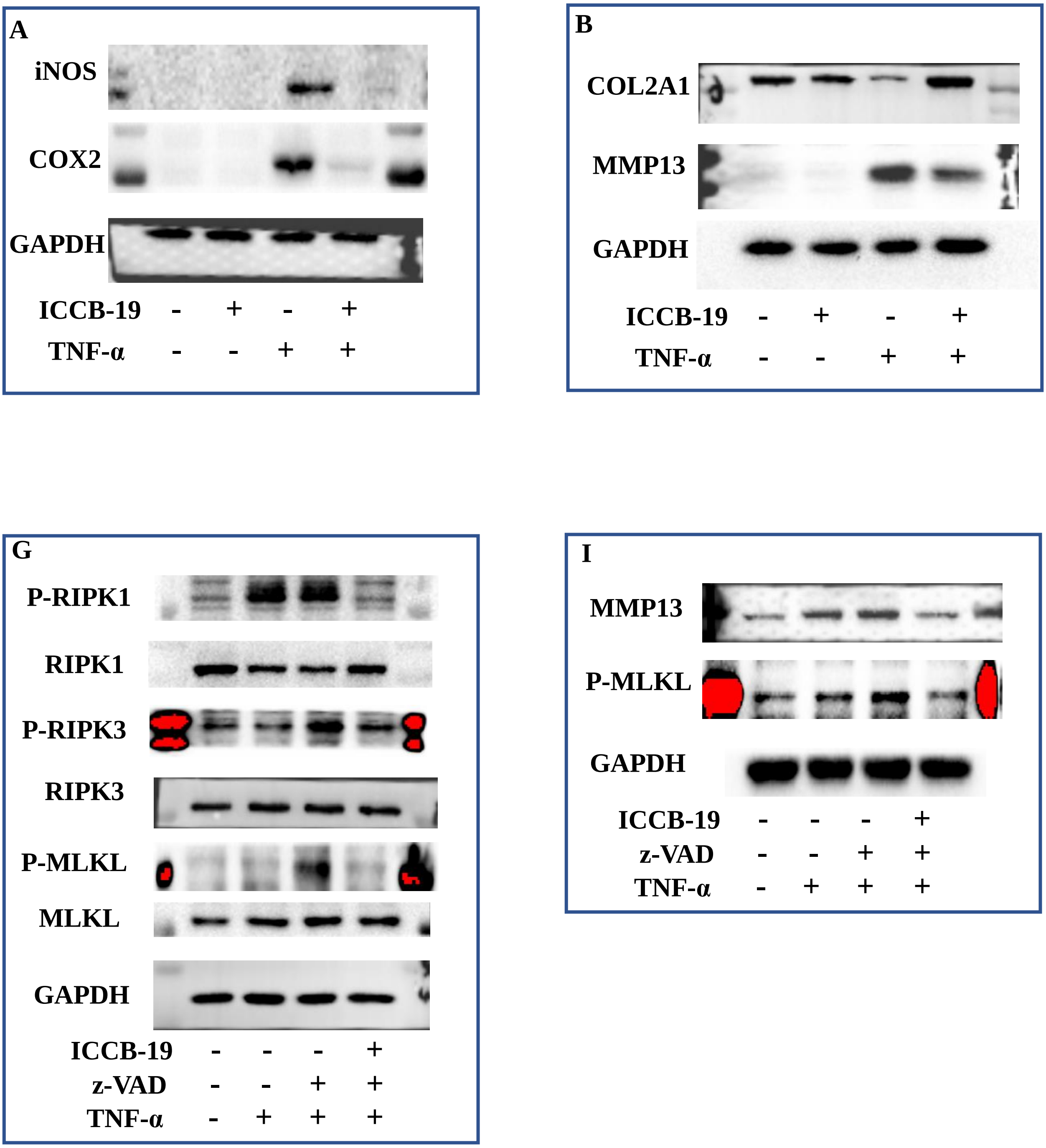
A
iNOS
COX2
GAPDH
ICCB-19 - + - +
TNF-α - - + +
B
COL2A1
MMP13
GAPDH
ICCB-19 - + - +
TNF-α - - + +
G
P-RIPK1
RIPK1
P-RIPK3
RIPK3
P-MLKL
MLKL
GAPDH
ICCB-19 - - - +
z-VAD - - + +
TNF-α - + + +
I
MMP13
P-MLKL
GAPDH
ICCB-19 - - - +
z-VAD - - + +
TNF-α - + + +

Fig.7

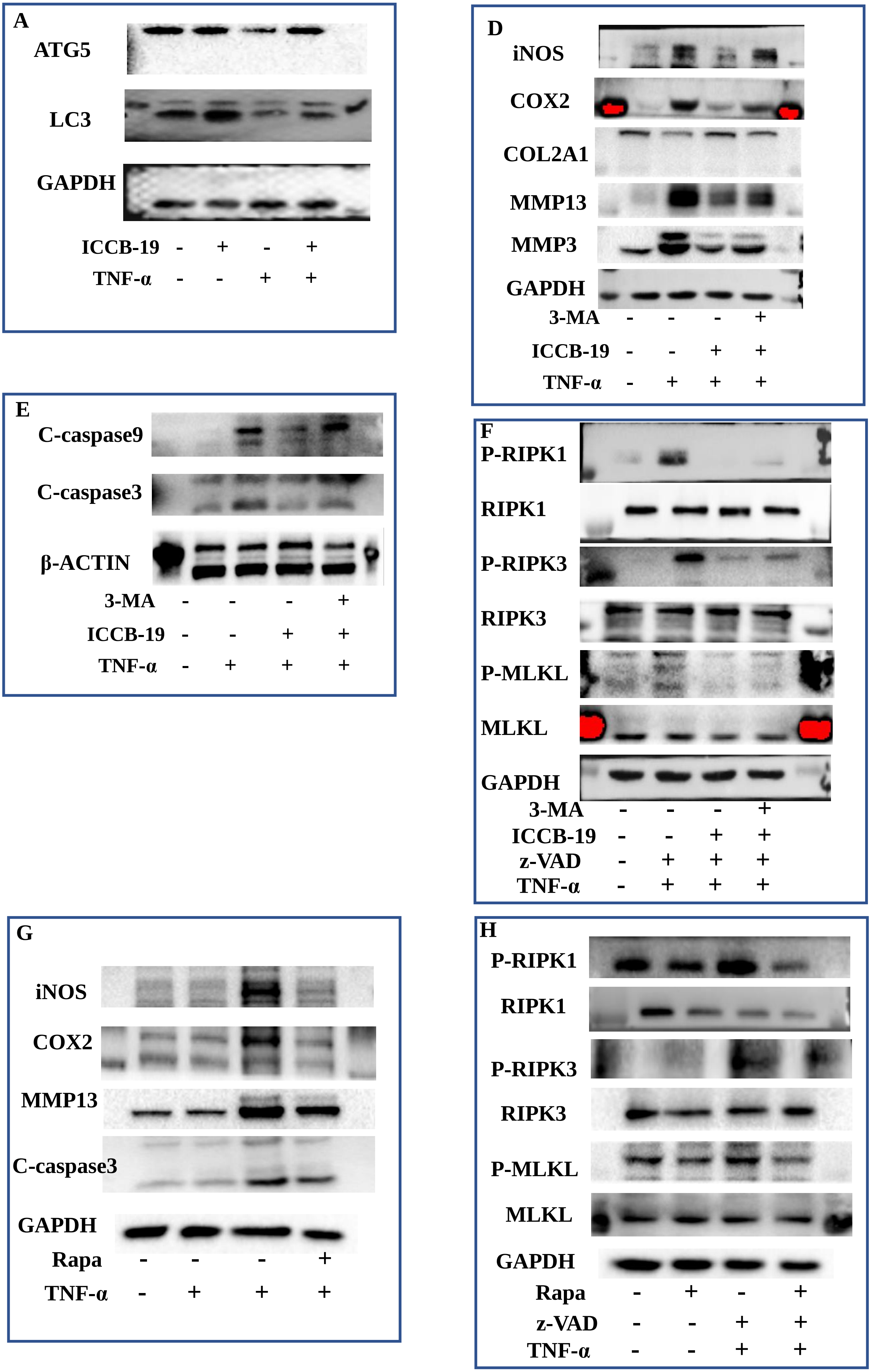
A
ATG5
LC3
GAPDH
ICCB-19 - + - +
TNF-α - - + +
D
iNOS
COX2
COL2A1
MMP13
MMP3
GAPDH
3-MA - - - +
ICCB-19 - - + +
TNF-α - + + +
E
C-caspase9
C-caspase3
β-ACTIN
3-MA - - - +
ICCB-19 - - + +
TNF-α - + + +
F
P-RIPK1
RIPK1
P-RIPK3
RIPK3
P-MLKL
MLKL
GAPDH
3-MA - - - +
ICCB-19 - - + +
z-VAD - + + +
TNF-α - + + +
G
iNOS
COX2
MMP13
C-caspase3
GAPDH
Rapa - - - +
TNF-α - + + +
H
P-RIPK1
RIPK1
P-RIPK3
RIPK3
P-MLKL
MLKL
GAPDH
Rapa - + - +
z-VAD - - + +
TNF-α - - + +
